# Strategies to Reduce Maternal Death Rate and Improve the Provision of Quality Healthcare Services in Selected Hospitals of Vhembe District Limpopo Province

**DOI:** 10.3390/nursrep13030107

**Published:** 2023-09-11

**Authors:** Tshisikhawe Mahada, Takalani G. Tshitangano, Azwinndini G. Mudau

**Affiliations:** Department of Public Health, Faculty of Health Sciences, University of Venda, Thohoyandou 0950, South Africa; tshisikhawe01@gmail.com (T.M.); takalani.tshitangano@univen.ac.za (T.G.T.)

**Keywords:** maternal care, mortality rate, quality, services, strategies

## Abstract

The maternal death rate remains high in South Africa despite the availability of many existing strategies to improve the quality of service. A 30% increase in the maternal mortality rate was reported between 2020 and 2021, with Limpopo ranking fourth highest out of nine provinces. The Vhembe district maintained its position as the third-ranked area with a notably elevated maternal mortality rate. This study aimed to explore the strategies used to reduce the maternal death rate and improve the provision of quality maternal healthcare services based on participant experiences in selected hospitals of the Vhembe district, Limpopo Province. One hospital was purposively sampled from every four municipalities. A qualitative approach using a phenomenological design was employed. Twenty-eight participants were sampled using a convenience sampling method. The semi-structured interview guide was used to collect data, complemented by the use of an audio recorder, continuing until data saturation was reached. Data saturation was reached at the 20th participant; however, the researcher continued until 28 sampled participants were interviewed. An interpretative phenomenological analysis was used following the analytical stages of interpretative data analysis. The study proposal was ethically cleared by the University of Venda Ethics Committee (FHS/22/PH/08/3108). The results indicate that, despite implementing strategies to improve maternal healthcare services and reduce the maternal death rate, several factors, such as the lack of material resources, shortage of staff, incompetent staff and poor infection control, affect the quality of maternal health services in the Vhembe district. The Limpopo Department of Health and hospital management should ensure that hospitals have all the necessary resources and support healthcare professionals through in-service training to ensure the functionality of existing strategies.

## 1. Introduction

Maternal death resulting from complications between pregnancy and childbirth remains a global public health systems challenge [1]. Approximately 287,000 women died worldwide during childbirth in 2020. Out of 287,000, 800 women died every day in 2020 due to preventable causes associated with pregnancy and childbirth globally [2]. Nearly 95% of maternal deaths occurred in low- and middle-income countries in 2020. Countries have unified to quicken the decline in Maternal Mortality Rate (MMR) by 2030 to reach the Sustainable Development Goal (SDG). In low- and middle-income countries, a high MMR has been associated with several systematic factors, which include inadequate facilities, a difficult access to care, shortage of practitioners with necessary training at various skill levels, inappropriate treatment, medication faults and incorrect diagnosis [3]. Thousands of women die yearly in low- and middle-income countries due to pregnancy-associated causes [4]. In Sub-Saharan Africa, women are at risk of losing their lives as the MMR is 47 times higher compared to women in America [1]. Enormous efforts have been made to improve quality services in different countries across the world. Guidelines, standards, policies and strategies have been implemented for healthcare professionals to follow as standardized practices that should be followed in healthcare facilities [5].

One of the strategies implemented to reduce the MMR and improve the provision of quality healthcare services is ESMOE (Essential Steps in the Management of Obstetric Emergencies), which establishes essential steps in managing obstetric emergencies [6]. ESMOE was implemented as an action plan to reduce increasing rates of MMR by building the clinical staff’s self-confidence and capacity to produce desirable results in maternal care in 2008. Moreover, BANC (Basic ANtenatal Care) was also implemented in 2008 to reduce the MMR and improve the MHC (Maternal Health Care) service provision targeting the SDG by 2030 [7]. BANC was introduced as a quality improvement strategy based on the belief that good-quality antenatal care could reduce the MMR. CARMMA (Campaign for Accelerated Reduction of Maternal Mortality in Africa) was also implemented in May 2012 to lower the unsatisfactorily high maternal and child death rate in SA through promoting early bookings and antenatal class attendance [8]. Additionally, ENAP (Every Newborn Action Plan) was launched in 2014 with the aim to provide a road map of strategic actions for ending preventable stillbirths and newborn mortality [9].

The World Health Organization (WHO) has reported that there has been a minor improvement in reducing the MMR and related complications worldwide because the MMR decreased by 38% from 342 deaths to 211 deaths per 100,000 live births in 2018 [10]. This decrease means that the annual proportion of reduction is 2.9%. However, the annual average is less than half of the percentage needed to meet the SDG, which is estimated to be 70 maternal deaths per 100,000 live births. Despite minor progress in reducing maternal deaths in South Africa (SA), deaths due to complications during pregnancy remain high, evidenced by 26 deaths per 100,000 live births [11]. Furthermore, in SA, thousands of women die resulting from birth-related conditions such as prolonged and obstructed labor, unsafe abortions and infections during their stay in hospitals [12].

It was generally acknowledged that the MMR is unacceptably high in SA (South Africa) [13]. A report covering 2019–2021 rates revealed that 80% of women who did not make it during childbirth received care in district hospitals where critical care, emergency medical services and specialists may not be available [2]. Though the MMR declined from 150 deaths per 100,000 births to 113 per 100,000 live births in 2019 in SA, an increase was reported between 2020 and 2021 [14]. Out of nine provinces of SA, deaths were high in Limpopo, Free State, Eastern Cape and KwaZulu-Natal. SA is dedicated to addressing concerns about inequalities through the implementation of strategies. Furthermore, the MMR was reported to be declining gradually by only 2.6% yearly, but this was far from the annual decline of 5.55% required to achieve the SDG [13]. Moreover, an increase of 30% in maternal deaths during the COVID-19 lockdown was reported. The national target is that the MMR should not be more than 140 deaths per 100,000 live births [7].

Regardless of all the efforts by the Department of Health, it is evident that there are still gaps, and additional work still needs to be accomplished [15]. A poor MHC service continues to be a significant concern. Moreover, most maternal deaths can be prevented, as healthcare policies, standards and strategies are well known. The main contributing factors were the lack of resources, including human resources and disrespect. Abuse in South African MHC services was labeled as one of the country’s greatest ignominies. Physical abuse, verbal abuse, non-confidential care and neglect were also included [2].

Limpopo was classified under some provinces with a high MMR between 2020 and 2021 [16]. Non-pregnancy-related conditions were reported to be part of the causes of maternal deaths in Limpopo, wherein 17.6% were obstetric hemorrhage, 10.2% were pre-existing medical and surgical disorders and 5.7% were anesthetic complications [17]. Additionally an increase in MMR in the Vhembe district was reported by saved mothers in 2018. Despite the government’s effort to reduce the MMR, between 2016/17 and 2018/19, there was an increase in MMR instead of a decrease in Vhembe, which emphasizes a challenge in the MHC services district [16]. Although, in 2019/2020, Vhembe’s MMR decreased to 66.5%, it remains among the top three districts with a high MMR in Limpopo Province. Against this background, this study emerged to explore strategies to improve the quality of MHC and reduce the MMR based on participant experiences in selected hospitals of the Vhembe district, Limpopo Province. Therefore, if the MMR remains high in the Vhembe district regardless of the implemented strategies, then what will work to reduce Maternal Mortality Rate and improve the quality of MHC service provision?

The following objectives were achieved:

- To explore the experiences of patients and healthcare professionals regarding the maternal healthcare services provided in selected hospitals.
- To describe factors affecting the provision of maternal healthcare services grounded on participant experiences in selected hospitals.
- To explore views of midwives and doctors providing MHC services about existing strategies to reduce the MMR and improve maternal health provision substantiated by their experiences.
- To explore perceived strategies to improve maternal healthcare services and reduce the MMR.

## 2. Materials and Methods

### 2.1. Study Design

A qualitative interpretative phenomenological design was used to explore the strategies to reduce the MMR and improve the provision of quality MHC services, focusing mainly on midwives, doctors and pregnant women’s experiences of maternal healthcare services in selected hospitals. This design was also utilized to understand the phenomenon of the level of quality of MHC services in selected hospitals given existing strategies. The phenomenological design was more appropriate for this study because it assisted the researcher in gaining insight into patients’ experiences regarding the maternal care they receive in hospitals. In addition, this design enabled the unpacking of the efficacy of existing strategies of improving MHC and reducing the MMR in selected hospitals of the Vhembe district. The researcher had background information regarding maternal healthcare services in the Vhembe district. However, this was not used in any form, to ensure resonance and accuracy of the results. The integrated guidelines for reporting qualitative studies (COREQ) [18] were utilized to generate the sections underneath, displaying how this paper addresses issues of rigor, i.e., how participants were engaged, depiction (whether the accurate participants were engaged) and reflexiveness (whether participants were engaged in a meaningful, equitable and ethical manner).

### 2.2. Setting

This study was conducted in four sub-districts (Thulamela, Makhado, Collins Chabane and Musina) of the Vhembe district, Limpopo Province. The Vhembe district is one of the five districts of Limpopo Province in South Africa. The country’s northernmost district shares its northern border with the Beitbridge district in Matabeleland, South Zimbabwe. The Republic of Zimbabwe surrounds the Vhembe district to the north, Mpoani (DC33) to the South, Capricorn (Dc35) to the Southwest and the former Venda. Data were collected in four hospitals (1 per district). Hospitals in the Vhembe district were selected because it was reported on district health barometer (DHB) that despite the government’s effort of implementing various strategies to reduce the MMR, between 2016/2017 and 2018/2019, there was an increase in MMR instead of a decrease in the Vhembe district (DHB, 2020), which emphasizes that there are challenges in MHC services (health system institute). Additionally, despite a decrease in 2019/2020, the Vhembe district still falls under 3 districts with a high MMR in Limpopo Province. A non-probability purposive sampling technique was utilized to sample hospitals. The researcher selected hospitals based on their judgement through reading news and reports about the kind of MHC services provided. The convenience sampling method was used to sample midwives, doctors providing maternal health services and pregnant outpatients. Twenty-eight participants formed part of this study, including three midwives, one medical doctor and three pregnant outpatients per hospital. The first author recruited participants to participate in this study. Out of all the participants, no one withdrew until the end of this study.

### 2.3. Data Collection

The first author (an MPH student on research training) conducted the interviews. Interviews were conducted in a quiet room occupied by one participant at a time and the first author. A rapport was built for flawless interaction with the recruited participants. In-depth interviews were used to collect data from participants who specified their willingness to participate in this study by signing consent forms. The consent forms were signed after revealing information about the first author. That comprised researchers’ characteristics such as interest in the topic, the aim of this study and personal goals. Ethical clearance from the ethics committee of the University of Venda was obtained (FHS/22/PH/08/3108). Approval for data collection from the Limpopo Department of Health, the Vhembe health district and four selected hospitals was obtained. Data were collected for three weeks in January 2023. The interviews were guided by an interview guide encompassing three central questions: *What are your views regarding the maternal health care services provided in selected hospitals? In your opinion, what factors affect the provision of maternal healthcare services in selected hospitals? What are your views regarding the existing strategies to reduce MMR and improve maternal health provision? What should be done to improve maternal healthcare services and reduce MMR*?

Probing questions were asked; however, they depended on how participants responded to the central questions. Central questions were formulated in English and, during the interviews, they were explained in Tshivenda and Xitsonga to those who needed help understanding. Pretesting was conducted to test and validate the research instrument and identify any possible errors for improvement during data collection for the main study. Pretesting was performed in one of the hospitals that had the same characteristics as the sampled hospitals. Midwives, doctors and pregnant outpatients who met the inclusion criteria were included in pretesting. The researcher realized that the questions were not clear enough, because participants were not responding in relation to the research topic and the aim of this study. Furthermore, the researcher encountered challenges in probing, which led to the need for rephrasing the questions. Participants in the pretesting did not form part of the main study. Participants allowed the researcher to use audio recorders, which captured data as a backup to field notes taken throughout the interviews. The main reason for recording was to ensure that no critical information was missed. Each interview lasted for 30–40 min.

### 2.4. Data Analysis

Data were analyzed using the interpretive Phenomenological Analysis (IPA) method to ensure coding and organizing emergent themes. IPA was chosen because it examines how individuals make sense of their lived experience and the researcher was more interested in gaining insights into participant experiences in relation to maternal health services. The researcher followed the analytical stages of interpretative data analysis by Smith (2012), namely Stage 1: multiple reading and making notes, Stage 2: transforming notes into emerging themes, Stage 3: seeking relationship and clustering themes, and Stage 4: writing up an IPA study. The researcher read the transcripts several times to understand the data. Additionally, each audio recording was played multiple times to ensure that every fact was noted down. The researcher made certain notes regarding the conducted reflections and observations during the interview. The researcher emphasized notes made during the first analytic stage rather than focusing more on transcripts. The researcher formulated a transitory phrase at a substantially advanced level of simplification or concept, which referred to a stronger healthcare conceptualization. In addition, the researcher coded data into superordinate themes and then developed a way of connecting all the themes that appeared. Transcripts were coded manually. The researcher clustered themes according to conceptual comparisons. Connections across themes were identified before the following participant’s transcripts and recordings were addressed. The clusters were assigned descriptive tags. Furthermore, themes that did not match well with the emerging construction, due to a frail evidential base, were not considered. Once all transcripts had been analyzed, outlines across transcripts were investigated and superordinate themes were created that captured the shared experience of the participants. Participants were provided with copies to ensure that the transcribed data accurately reflected what they said.

Therefore, the researcher wrote a narrative interpretation of this study. It comprised obtaining the ultimate outcomes of themes and then describing each one of them separately. The researcher used the interview excerpts and analytic remarks from the authors so that she could provide examples and outline the themes. However, the researcher kept their views to themselves through the analysis.

### 2.5. Trustworthiness

Four measures to ensure trustworthiness were followed: credibility, transferability, dependability and conformability. Credibility was ensured through prolonged engagement with data collected and member checks, where transcribed data were sent to participants to check accuracy and resonance. Furthermore, the researcher presented the inquiry findings to supervisors (co-authors) to obtain remarks. Transferability was ensured by collecting detailed information and a thick description of the data. The researcher gave a thick description by elucidating all the research methods beginning with data gathering, the setting-up of this study until the making of the final report. Furthermore, the researcher provided a thick description by presenting adequate information about the researcher as an instrument, participants/respondents and researcher–informant relations to allow the reader to choose how the results may be transferred. To ensure dependability, a detailed description of research methods was provided. Audit trail was also used to ensure dependability where the researcher clarified all the research choices and activities to demonstrate how data were gathered, recorded and analyzed. The researcher of this study accomplished that by vigilantly monitoring the evolving research approach and keeping an audit trail, explaining the order of research activities and procedures, data compilation influences and analysis, and developing themes and sub-themes. The researcher also ensured dependability by saving consequent documents for double-checking the research procedures, interviews, raw information, manuscripts and records gathered from the field. Information collected during the interviews was recorded and transcribed without any changes, to ensure conformability. Lastly, to ensure confirmability, notes were retained in a secure location to create an acceptable trail and determine conclusions and interpretation.

## 3. Results

Table 1, Table 2 and Table 3 represent the biographical information of participants.

Data saturation was reached at the 20th participant. However, the researcher continued with data collection to discover the experiences of sampled participants in the remaining hospital, because participants were sharing their experiences based on the hospitals they were providing or receiving care from. Four superordinate themes were identified that brought together the data from all 28 participants (see Table 4). Three themes, one, two and three (experiences in maternal health services, factors affecting maternal health services, and recommended strategies to improve maternal healthcare and reduce MMR), were identified in all 28 transcripts. Moreover, theme three (views of existing strategies/approaches) was identified in 16 transcripts, which entailed healthcare professionals only. However, some transcripts needed to be more evident. The researcher offered participants an opportunity to reflect on the care they have been receiving (patients) and the care they have provided (midwives and doctors). Some insights contributed to the creation of superordinate themes and sub-themes. Table 4 below presents superordinate themes and sub-themes that emerged from the data analysis.

### 3.1. Theme 1: Negative Interactions with Midwives

All 28 study participants narrated different experiences when asked *May you kindly share your experience of maternal health services rendered in this hospital?* Shared experiences led to poor maternal health services or maternal death. The following are subthemes that emerged from their experiences:

#### 3.1.1. Negative Attitude of Midwives toward Patients

Four participants (P17F31, P20F30, P24F38 and P27F40) revealed that they had experienced negative attitudes in the form of anger and demotivation from midwives. One (P19F31) participant emphasized the issue of annoyance and ignorance and specified that she could see anger in the midwives’ faces. Therefore, she could not ask for help, potentially giving rise to complications and undesirable outcomes, including the possibility of death. Some of the experiences shared are as follows:

*“After giving birth, they instructed me to go to the bathroom, I was heavily bleeding and when I got out of the bathroom there was blood everywhere. They shouted at me for using a certain bathroom, which was the closest. I apologized and they both gave me an attitude and looked at me in a bad way as if they were disgusted”* (P24F38).

Participant P17F31 said

*“I felt disrespected because they did not involve me in anything, I just found myself in the theatre ward for C-section without explanations. Instead of informing me that I had complications, they were just going up and down, ignoring me as if they could not see me”.*

*“When you fail to do something, they will ditch you and tell you to assist yourself because you know better”* (P20F30).

#### 3.1.2. Non-Empathetic Language of Midwives toward Patients

Most pregnant outpatients in this study revealed that midwives used non-empathetic language accompanied by threats, scolding and rude approaches. One participant (P21F32) said they mistreated them, especially if they had complications. She added that, during the examination, they did not use dignified language; they used harsh tones and shouted. The following are some of the experiences of participants:

*“I experienced the worst with my second born in this hospital. I hope it will not happen again. I was slapped and the nurse was harsh to me, using inappropriate language and telling me to push the same way I made my baby. They do not respect us at all, I arrived here for my check-up at 7, but I will leave here at 1 and when you ask them, they normally respond harshly… hmm that’s all I can say”* (P21F32).

*“They shouted at me until I did not know what to do with myself”* (P23F20).

#### 3.1.3. Frustration among Nursing and Medical Staff

Two participants (P20F30 and P22F23) disclosed that healthcare professionals were always upset. They disclosed that midwives became frustrated to such an extent that, when patients asked for help, they shouted instead of attended to them. Moreover, participants revealed that healthcare professionals failed to maintain effective communication. The following narratives evidence this:

*“This will be my second time giving birth in this hospital. The experience was not that bad or good. I gave birth here in 2021, it was my first time, but the nurse that was helping me kept on leaving me alone on my own. When I told her that I was in pain, she would tell me to stop making noise because I was not the only patient in the ward. She added to say that “we are only two here and we have two hands each, appreciate the little that you are getting”. Nurses were not enough that day, if I had money, I was going straight to a private hospital”* (P4F29).

*“Midwives will shout at you from the beginning of their shifts to the end. They kept on shouting at me because I could not get some of the instructions right as I was a first mother and in pain. You could see the midwife’s frustration on her face. Later, she stopped talking to me and just performed examinations without talking to me”* (P19F24).

*“Some midwives even instruct patients to do certain things without monitoring them closely while they are busy with their phones. I cannot wait for this week to end”* (P13M34).

#### 3.1.4. Ill-Treatment of Patients by Midwives

The majority of patients reported mistreatment by healthcare professionals. Participants reported that they were being left unattended and unsupported. It was also disclosed that midwives insulted and physically assaulted them, which was a violation of their human rights (P18F33). The following are some of the participants’ experiences.

*“I gave birth to twins in 2021 in this hospital, I was the youngest in the ward they called an older lady saying that she should come and deal with me. She kept on slapping my thighs and pinching me, I still have marks on my thighs”* (P18F33).

*“They were slapping a lady next to me, threatening her that if she does not push harder, they will lose her and the baby, I was scared for my life”* (P17F31).

#### 3.1.5. Lack of Information

Most participants specified that there was a lack of information on maternal health. Two participants (P20F30 and P25F26) emphasized that they wanted more from the information and explanations provided by the midwives. One participant (P23F20) said that the midwives were more interested in having patients comply with their demands than allowing them to ask questions for clarity. The following narratives evidence this:

*“When I gave birth for the first time my new-born baby was kept here for 3 days. They did not tell me the reasons. I asked one of the nurses she said I should ask the doctor and the following day a new doctor came in; she told me that she needs to examine the baby as her call started that day. They discharged me after three days without knowing what the problem was. As I continued attending monthly check-ups after four months, I found out that my baby has a disability called cerebral palsy”* (P20F30).

*“I have been coming here for two months for check-ups because of some complications. They did not give me enough information on where to go to attend antenatal classes. I did not even know about those classes. I heard some ladies talking about them on the queue until I asked them. When I ask the nurse, she said I should be responsible enough to know what is expected of a pregnant woman in this hospital”* (P23F20).

*“Nurses just do procedures without prior explanations for me to know what is happening or what to expect. I remember in 2016 they just directed me to take off my clothes without clarity”* (P26F26).

*“I was referred from a clinic as a high-risk patient, I spent 4 h without anyone coming to me to explain what was happening and the cause of the delay. They did not even examine me or monitor me. After 4–5 h, I was directed to sit on a wheelchair without explanations and boom, I had a child via C-section”* (P18F33).

### 3.2. Theme 2: Factors Affecting Maternal Care Provision in Participating Hospitals

Based on participants’ experiences, all 28 study participants pointed out factors that led to poor maternal health and maternal deaths in selected hospitals. Most participants disclosed that maternal health services and some deaths occurred due to a lack of material resources, shortage of staff, incompetent staff and poor hygiene control. This observation is depicted in the following sections:

#### 3.2.1. Shortage of Resources

##### Category 1: Shortage of Materials

Many participants highlighted that the insufficient material resources in hospitals had a direct impact on the maternal health system. Two participants (P13M34 and P15M40) openly stated that they used manual monitoring devices in a unit full of patients, resulting in the delayed detection of conditions such as hemorrhage, which leads to death. Furthermore, midwives emphasized the shortage of birthing beds, where one participant (P4F29) indicated that they were forced to help patients on the floor due to the shortage of birthing beds. Moreover, pregnant women complained about water and disclosed that they sometimes did not bathe, because of the water, and the toilets were mostly not user-friendly, due to water shortages. The following are some of the statements by participants:

*“It is stressful to work in a hospital without proper equipment. I have been working in this hospital for three years, but we do not have proper equipment in the emergency room and it puts our patients at risk as we must use what we have. We order every financial year, but there is no change and their excuse is funding”* (P13M34).

Participant P4F29 said:

*“The government is failing us; we need more birthing beds. We need more of those. Women are giving birth on the floor and logging complaints directed to us. We have no control over some things here, ours is to render our services and nothing more. But one thing for sure anything can happen while a woman is lying on the floor”.*

*“I was admitted for three days only bathed two days and the other day there was no water at all. They discharged me that day and I bathed when I got home”* (P24F38).

##### Category 2: Shortage of Resources

All 28 participants were unhappy about the lack of human resources, which was pointed out as a significant contributing factor leading to adverse outcomes in maternal healthcare services. Moreover, one participant (P9F30) revealed that it undoubtedly increased the rate of deaths and dissatisfaction from patients. Both parties shared their experiences concerning the shortage of staff; the following narratives were shared:

*“I will take you back to when I started working in this hospital in 2020. I was assigned to a unit which had only two midwives and the other one had recently started working, she was not that experienced. The unit was full of patients and they all required adjacent monitoring and assessments for positive outcomes. Because of shortage of midwives, I had to assist the majority of patients alone and as a result, one patient ended up losing her baby girl as she delivered twins, a boy and a girl. If we had enough midwives, we would have saved that baby”* (P14M38).

Participant P28F31 added to say:

*“This will be my second time giving birth in this hospital. The experience was not that bad or good. I gave birth here in 2021, it was my first time, but the nurse that was helping me kept on leaving me alone on my own. When I told her that I was in pain, she would tell to stop making noise because I was not the only patient in the ward. She added to say that “we are only two here and we have two hands each, appreciate the little that you are getting”. Nurses were not enough that day, if I had money, I was going straight to a private hospital”.*

Another participant indicated that

*“I would say there are no changes with the experience that I had while I was still a student, when I gave birth and now as I am working here. I did my practical in this hospital in 2018, I used to take 15-min breaks because the units were always full and the workload was just too much. There was shortage of stuff and as students, we ended up doing some assessments without supervision. Laughter… in 2019, I gave birth here and I assisted myself somewhere somehow because I knew the challenge and if I did not my child would have died. Even now I am assigned with an older lady she is not that active; I am doing everything alone”* (P2F29).

#### 3.2.2. Poor Infection Control

All categories of healthcare professionals emphasized that infection control was poor due to a shortage of sterilizers. Two midwives (P1F52 and P6F42) revealed a water shortage in hospitals, making it difficult to prevent infections as they always had to wash their hands with warm water. One midwife (P8F56) said it was difficult to prevent infections because days would pass without water for pregnant women to bathe. The following statements support this:

*“We have water problems and this has been happening for over a year, so we find it difficult to prevent infections as we need water for that”* (P8F56).

*“The department should ensure that we have all the resources, most especially water, because sometimes patients are forced to fetch water themselves and if we need to sterilize some equipment, we have to go to the nearest hospital to perform that and that tend to put patients in danger most especially if they need to be attended urgently”* (P1F52).

##### Theme 3: Views on Existing Strategies

Participants were asked the following question, *what is your view regarding existing strategies to reduce maternal death rate and improve Maternal health service provision?* All 16 healthcare professionals shared their views about existing strategies implemented to improve the quality of maternal health services and reduce the maternal death rate. They shared their opinions on how they perceived those strategies. They further specified if they were effective or not.

### 3.3. Theme 3: Views of Healthcare Professionals Regarding the Existing Strategies to Improve Maternal Care

#### 3.3.1. ESMOE (Establishing Essential Steps in Managing Obstetric Emergencies) Formation

All doctors who formed part of this study revealed that ESMOE expands affordability and the standard of treatment for pregnant women undergoing obstetric emergencies as it shapes their confidence to function effectively. One midwife (P6F42) disclosed that it is effective. However, they must work as a team in maternity units to produce desirable results. The following were some of the narratives from participants:

*“My view about ESMOE is that it is effective and valuable in the sense that it enhanced our knowledge and skills about maternal health services. Even though there is a modest improvement in the functionality of this hospital it has helped us in preventing direct causes of maternal deaths caused by preventable causes such as eclampsia and postpartum hemorrhage. It can also be more effective if we work as a team as healthcare professionals”* (P6F42).

*“ESMOE is essential, the department came through for us (laughter). It is very important as it can be used as an intervention in many cases, but some of the midwives in this hospital lack knowledge and they wait for the doctor to do everything and some areas are part of their duties”* (P16F31).

Another participant added to say that

*“This strategy is very helpful, but I believe that midwives need to be provided with relevant guidelines for maternity care and essential steps in the management of obstetric emergencies. I have realized that challenges are being experienced by midwives in the execution of their roles as they interface with the healthcare team”* (P13M34).

#### 3.3.2. CARMMA (Campaign for Accelerated Reduction of Maternal Mortality in Africa) Implementation

Most healthcare professionals from both categories (midwives and doctors) were familiar with this strategy. They highlighted that CARMMA is a very effective strategy that enables them to promote early antenatal care, booking and attendance of pregnant women. Moreover, they had different opinions about its effectiveness in patient adherence and willingness to make it work, especially in primary healthcare. This is what some of the healthcare workers said:

*“CARMMA makes things easy for us as it advances access to skilled birth attendants and that on its own improves child survival. But it would have been more effective if nurses in primary healthcare refers patients on time or let me just say if obstetric ambulances arrive on time with patients. Late arrival affects CARMMA’s effectiveness as it aims to improve skilled birth by us (doctors and midwives)”* (P14M38).

Participant P3F35 indicated that

*“As much as CARMMA advocates for ‘no woman should die while giving birth’, we are trying our best to plan campaigns. My view about CARMMA is that it is helpful and effective because it encourages us to provide women with public information. We have midwives and health promoters teaching pregnant women about maternal health, what to do and what not to do. And I believe that if we continue like this, we are going far as a hospital”.*

#### 3.3.3. BANC (Basic Antenatal Care) Establishment

All 16 healthcare professionals specified that BANC is an essential strategy as it provides them with guidelines and knowledge to perform antenatal care effectively. Three midwives (P1F52, P3F35 and P5F31) highlighted that it helps them notice complications and preventable sicknesses in mothers and children on time.

*“My view about BANC is that it is the best strategy that the department has ever implemented. This strategy is operational as it aids pregnant women with answers that they have about pregnancy most especially first-time mothers. Pregnant women receive health advice and guidance and that makes things easier for us as midwives”* (P5F31).

*“BANC is an essential strategy that ease maternal health service provision for us. It helps us to identify high-risk cases and come up with suitable interventions. We urge pregnant women to attend these antenatal care classes because it enables us to prevent the development of complications”* (P3F35).

*“My view about this strategy is that it is the greatest approach to prepare the family for the coming baby. BANC is beneficial, if women attend those classes it helps to lessen the stress and uncertainties of pregnant women concerning delivery process most especially first-time mothers. Those interactions and discussions help mothers to understand their states. I really enjoy antenatal classes”* (P1F52).

#### 3.3.4. ENAP (Every New-Born Action Plan) Approach

Not all healthcare professionals were familiar with ENAP. Only one doctor (P15M40) and five midwives (P5F31, P8F56, P9F30, P10F31 and P12F27) were familiar with it. One midwife (P5F31) emphasized that ENAP serves its purpose of quality maternal care improvement as they plan different interventions to achieve its purpose through ENAP. However, one doctor (P15M40) believed there is a need for the periodic monitoring and reporting of progress concerning the ENAP approach. The following statements evidence this:

*“Since the launch of ENAP, the district and hospitals planned actions and interventions like neonatal resuscitation and thermal care, but we do not get reports specifically for those interventions. So, my view about this strategy is just neutral. But if I am to vouch for us as healthcare professionals, I would say its working”* (P15M40).

*“ENAP led to progress in reducing maternal deaths rate. With the launch of this strategy, I feel more optimistic than ever about the future of new-borns and their mother’s health”* (P5F31).

### 3.4. Theme 4: Perceived Strategies to Improve Maternal Healthcare and Reduce MMR

Participants were asked *What should be done to improve the provision of maternal healthcare services and reduce MMR in this hospital*? All 28 participants had various suggestions that can be implemented to reduce the maternal death rate and improve the quality of maternal health services. The following are the participant’s main recommendations:

#### 3.4.1. Continuing In-Service Training

Eleven participants (P2F29, P6F42, P8F56, P9F30, P10F31, P11F25, P12F27, P13M34, P14M38, P15M40 and P16F31) highlighted that in-service training should be prioritized to enable healthcare professionals to provide quality services. Two midwives (P8F56 and P11F25) believed that continuing in-service training will improve their skills and knowledge in maternal health. One doctor (P14M38) highlighted that skilled and competent healthcare professionals can achieve the reduction in MMR; therefore, they need in-service training. Participants stated the following:

*“There is a gap in midwives in-service training, CTG results sometimes confuses me. I find it hard to record the baby’s heart rate and the mother’s contractions. I rely on my colleagues and that’s way too risky”* (P11F25).

*“In-service training should be provided as not all of us were not privileged enough to get caesarean workshop procedure… uhmm I still feel like I need more skills on conducting caesarean section procedure”* (P8F56).

*“If I did not take a refresher course while I was an intern as a doctor, I would not have known how to perform a caesarean section procedure”* (P14M38).

#### 3.4.2. Launching Maternal Outreach Services

One pregnant outpatient (P18F31) suggested that maternal outreaches through maternal health education in various communities could help to improve maternal healthcare providers and reduce the maternal death rate. She believed that, if those outreaches entail free pregnancy check-ups by midwives and doctors, there might be a noticeable change in the standard of maternal health. However, one doctor (P13M34) specified that he had provided outreach programs in primary healthcare facilities and urged other healthcare professionals to consider doing that. The following are some recommendations by participants:

*“Nurses and doctors need to find ways to teach us about pregnancy and what they expect from us. The clinic in my village is not functional at all and I stay far from the hospital and when I get here you will find that they are done with antenatal classes. So, if they visit communities at least once a month, we will not miss out”* (P17F31).

*“I visit two local clinics in this area once a month in the form of outreach programmes because I realized that they refer patients to the hospitals only to find that complications have escalated and they could have been prevented if attended on time”* (P13M34).

#### 3.4.3. Priority Equipment Provision

All doctors who formed part of this study suggested that the department and hospital management should ensure that all resources are available for safer care. One doctor (P13M34) pointed out that available equipment was inadequate and some did not function properly. Moreover, all midwives participating in this study believed that resources such as birthing beds and water should always be available.

*“The department should ensure that we have all the resources, most especially water, because sometimes patients are forced to fetch water themselves and if we need to sterilize some equipment, we have to go to the nearest hospital to perform that and that tend to put patients in danger most especially if they need to be attended to urgently”* (P4F29).

P13M34 indicated that

*“It is stressful to work in a hospital without proper equipment. I have been working in this hospital for three years, but we do not have proper equipment in the emergency room and it puts our patients at risk as we have to use what we have. We order every financial year, but there is no change and their excuse is funding”.*

#### 3.4.4. Employ More Healthcare Professionals

All 28 participants suggested that more healthcare professionals should be employed as that would serve as a strategy to improve the quality of maternal health services in hospitals. Two midwives (P2F29 and P12F27) highlighted that those retiring, passing away and resigning should be replaced to avoid work overload. This is demonstrated by the following statements.

*“More midwives and doctors should be hired; we are being overworked because we are understaffed that is the reason why we end up being demotivated…. uhhm, I’m not defending myself, but for us to be more productive, we need to be active and get enough rest”* (P5F31).

*“They need to hire more nurses because when we come here, we do not receive enough care as they will be rushing to others”* (P25F26).

*“They need to replace those that resigned, passed on or retired because the more they leave the more we become understaffed”* (P7F40).

## 4. Discussion

Negative interaction with midwives

The study findings revealed that patients encounter different experiences while receiving maternal care in selected hospitals of the Vhembe district, Limpopo Province. Some of the experiences they shared with the researcher are negative attitudes in the form of anger and demotivation from midwives. Patients refrained from seeking help due to annoyance and ignorance. This is similar to the findings of a study conducted in Bayelsa state in Nigeria that disclosed that negative attitudes by midwives outweigh positive attitudes as many women decide to seek care in the hands of unskilled, unexperienced and unqualified people as a way of avoiding negative attitudes in hospitals [19]. Similarly, a study conducted in Ikot Omin, Cross River Estate, Nigeria, revealed that the majority of participants who were pregnant women were not satisfied with the care they received during their pregnancy, because they reported negative attitudes concerning midwives [20]. Contrarily, a study conducted in three district hospitals of Kigali City, Rwanda, discovered that midwives have a positive attitude toward pregnant women concerning providing respectful maternity care. However, midwives reported that they face challenges such as work overload and a lack of labor monitoring materials, which affects their ability to provide respectful maternal care accompanied by a positive attitude [21]. It is evident that negative attitudes by healthcare professionals in African countries remain a challenge and, as a result, the standard of maternal healthcare remains poor. Consequently, women seek help from unskilled people, putting their health and children’s health in danger, resulting in maternal death.

This study also discovered that pregnant women experience a non-empathetic language approach by midwives. Most pregnant outpatients revealed that midwives use non-empathetic language accompanied by threats, scolding and rude approaches. This is consistent with a study conducted in a Midwife-Led Obstetric unit in the Tshwane district, South Africa, where women reported that midwives shout at them, label them, judge them and use rude remarks while addressing them [22]. This corresponds with a study conducted in the Ndola and Kitwe districts of Zambia, where participants indicated that they were verbally abused as they were scolded, shouted at and told hurtful words and displeasing statements and remarks [23]. Non-empathetic language is not only a concern in Limpopo Province. As a result, women become scared and confused during labor because of fears of being shouted at or judged, leading to the delayed detection of complications, poor maternal healthcare service and maternal death.

Pregnant women that formed part of this study disclosed that healthcare professionals are constantly upset and become frustrated to such an extent that when they ask for help, they shout instead of attending to them. Similarly, in a study conducted in Namibia, midwives admitted that they are constantly frustrated due to a shortage of staff and excessive workload, which leads to stress and burnout, especially during complicated labor [24]. A study conducted in Limpopo Province also discovered that due to an excessive workload, midwives experience physical exhaustion, anger and frustration, leading to poor maternal health services [25]. Due to feeling stressed and overwhelmed in the workplace, healthcare professionals are frustrated. Subsequently, some midwives have considered leaving the midwifery profession. Moreover, doctors consider opening their private practices or working for private hospitals, leaving public hospitals understaffed.

The study findings revealed that the majority of patients experience mistreatment by healthcare professionals. Participants reported that they were being left unattended and unsupported. In addition, patients disclosed that midwives insult and physically assault them, which explains the violation of their human rights. A study conducted in South African maternity settings reported that, globally, women experience ill-treatment in a pattern of physical abuse, verbal abuse, procedures without consent, neglect, non-confidential care and abandonment of care [26]. Similarly, a study conducted in Durban, South Africa, revealed that women described ill-treatment as verbal abuse from midwives, lack of privacy and midwives refusing to provide care [27]. By contrast, a study in Ethiopia uncovered no evidence of more systematic forms of mistreatment involving neglect and abuse by midwives [28]. Furthermore, healthcare professionals showed basic knowledge of patients’ privacy and consent. This shows that there is still a concern about the ill-treatment of patients by midwives in South Africa, as previous studies also discovered mistreatment that appeared as a form of abuse. Consequently, patients stopped or avoided going to hospitals where they experience mistreatment. Some patients open cases or sue those hospitals, which results in a staff shortage if the healthcare professionals involved get fired or arrested.

This study’s findings revealed a lack of information on maternal health in selected hospitals of the Vhembe district, Limpopo Province. Emphasis was made on the fact that participants were not satisfied with the information and explanations provided by midwives. They revealed that midwives are more interested in having patients comply with their demands than allowing them to ask questions for clarity. This corroborates with a study conducted in Middle Eastern countries in a narrative review where women reported dissatisfaction with the information and explanations provided by health professionals, as they specified that the information was insufficient and led to feelings of insecurity [29]. This is consistent with the findings of a study conducted in India, where it was reported that midwives perform vaginal examinations without any information or consent [30]. This shows that patients’ rights to information are being violated as healthcare professionals are expected to explain all procedures before implementation to allow patients to participate in the decision-making process. Therefore, patients report dissatisfaction, leading to poor treatment compliance and poor health outcomes.

Factors affecting maternal health services

This study’s findings revealed several factors affecting the maternal health system in selected hospitals of the Vhembe district, Limpopo Province. This study’s findings exposed a need for more essential materials, such as birthing beds, water and monitoring devices, which delay the possible detection of complications in labor wards. Similarly, a study by Mokoena [31] revealed the lack of material resources, electronic equipment and supplies, such as a glue meter for monitoring blood glucose and diagnosing meningitis, resulting in prolonged patient stays in the hospital. Furthermore, this corresponds with a study conducted by Musie, Peu and Pema [32], where midwives complained that there is a shortage of equipment assisting birthing women, such as birthing stools and birthing balls. On the contrary, Moyimane, Matala and Kekana [33] discovered that there is equipment in hospitals; however, it requires regular maintenance. A lack of essential resources blocks healthcare professionals’ ability to deliver quality healthcare services, leading to complications during labor and maternal death.

Staff shortage was identified as a significant contributing factor to poor maternal health services and maternal death. However, participants were specific enough to point out that there is a shortage of midwives and revealed that they end up providing poor maternal health services as they are understaffed. These findings concur with a study conducted in Malawi by Makhado et al. [34], stating that an increment in patient–nurse proportion in maternity units is associated with a shortage of midwives, where one midwife is anticipated to assess all pregnant women, which seems to compromise and lead to poor labor progress. According to a report published by (UNFPA), the World Health Organization (WHO) [10] and the International Confederation of Midwives (ICM) reported that the world is facing a shortage of about 900,000 midwives globally. On the contrary, a study conducted by Moyer et al. [35] discovered a need for more staff in various categories of healthcare professionals, i.e., laboratory technicians, doctors and emergency medical services, not only midwives. A shortage of staff severely affects the quality of maternal health as midwives are the backbone of maternal care. The more they are affected, the more maternal health services become poor because of an increased workload, leading to stress and burnout.

Poor infection control also contributes to poor maternal health and MMR. Study participants showed dissatisfaction with the state of infection control in hospitals. The findings discovered that there are no sterilizers, and water shortage continues to be a problem as they need water to prevent infections. These findings are consistent with a study conducted in Turkey, where the study’s main results revealed a poor infection control practice in hand hygiene, glove utilization and usage of bandages, resulting in hospital-acquired infection [36]. Similarly, a study conducted in South Africa by Maphumulo and Bhengu [37] revealed that patients and staff confirmed that some hospital departments had unacceptable physical environments, such as dirty toilets, to deliver quality healthcare. Patients find it difficult to wash hands, bathe and clean in the wards; consequently, it is difficult to prevent infections. Poor infection control is a challenge globally. Due to poor infection control, patients are more likely to acquire hospital-acquired infections, which might lead to severe complications in hospitals if detected late.

Views on existing strategies/approaches

The study participants had different views about strategies to reduce the MMR and improve MHC. They recommended ESMOE as a means to enhance both the affordability and quality of care for pregnant women experiencing obstetric emergencies. ESMOE is instrumental in bolstering the confidence of healthcare professionals in the field of midwifery, enabling them to perform their duties effectively. However, for this to be effective, healthcare professionals must work as a team in maternity units to produce desirable results. This finding is similar to the findings of this study conducted in 30 district hospitals from eight districts throughout South Africa, which revealed that ESMOE had improved the skill and knowledge of maternity healthcare providers [38]. On the contrary, a study conducted in KwaZululu-Natal discovered that despite the implementation of ESMOE, maternal mortality remains high in Kwazulu-Natal as it cannot be effectively implemented by all midwives [6]. This finding is in line with the findings of a study by Makhado, Mangena-Netshikweta, Mulondo and Olaniyi [8], which emphasized that irrespective of ESMOE implementation, more needs to be done for midwives and doctors to be able to manage obstetric complications and reduce maternal deaths in low- and middle-income countries. This study shows that if there is no teamwork, there will always be challenges with ESMOE utilization for healthcare professionals to manage obstetric emergencies, leading to poor maternal healthcare and maternal death.

The study findings emphasized that CARMMA is a very effective strategy enabling healthcare professionals to promote early antenatal care, booking and attendance of pregnant women. This finding corroborates with objectives outlined in a Northwest South Africa study that discovered that CARMMA has improved access to skilled birth attendants by allocating dedicated obstetric ambulances to every sub-district [39]. This finding also corroborates with the South African National Department of Health [40], which highlighted that CARMMA was implemented to lower the unsatisfactorily high maternal and child death rate in SA by promoting early bookings and antenatal class attendance. The study findings firmly emphasize that the attendance of antenatal classes and early bookings are essential features of quality healthcare services.

This study’s findings revealed that BANC is an essential strategy. It provides healthcare professionals with guidelines and knowledge to effectively perform antenatal care, helping them notice complications and preventable sicknesses in mothers and children on time. This finding is consistent with the South African Maternal, Perinatal and Neonatal Health policy implemented by the South African National Department of Health [9], which highlighted that through BANC, pregnant women who attend antenatal classes would be screened so that healthcare professionals can detect and prevent maternal complications that might occur before and after birth. The complete application of BANC will continue to produce desirable outcomes of maternal health services in hospitals as complications and preventable sicknesses will be detected on time.

This study discovered that most healthcare professionals who formed part of this study needed to be more familiar with ENAP. Furthermore, the findings underscored that ENAP is fulfilling its intended purpose, which is the enhancement of the quality of maternal care. Healthcare professionals are using ENAP to strategize various interventions aimed at improving maternal care. There is also a need for the periodic monitoring and reporting of progress with the ENAP approach. Contrarily, a study conducted in Iran discovered that ENAP is impractical because they identified holdups in maternal healthcare after analyzing a tool to identify obstacles delaying maternal care through ENAP [9]. Thus far, no studies have been conducted in SA to explore ENAP’s effectiveness. Additionally, in light of this study’s findings, it is essential to reintroduce ENAP and assess its effectiveness from the perspective of healthcare professionals, taking into account their views and comprehension of the program.

Recommended strategies to improve maternal healthcare and reduce MMR

This study discovered that various interventions could be executed to improve the quality of MHC services and reduce the MMR. Participants of this study suggested that there should be continuing in-service training to enhance midwives’ skills and knowledge for quality maternal health service provision. Similarly, a study in Ghana emphasized that midwives should be provided with in-service training to up-skill midwives’ knowledge to deliver healthcare services that would increase client satisfaction in childbirth care services in public health centers [35]. This finding is consistent with the findings of the scoping review that focused on Sub-Saharan Africa by Welsh, Hounkpatin, Gross, Hanson and Moller (2022) that to reduce maternal and neonatal morbidity, midwives should have access to “evidence-based in-service training materials” that comprise routine intrapartum care and antenatal care [41].

Launching maternal outreach services can improve maternal health service provision and reduce the MMR in Vhembe district hospitals. Participants suggested that maternal outreach through maternal health education in various communities, accompanied by free check-ups, could help improve MHC provision and reduce the MMR. This finding validates the findings of the study conducted in Ethiopia that community outreach services should be implemented to improve the knowledge of pregnant women about their condition and reduce the development of complications [29]. Findings of a study conducted in Gert Sibande in Mpumalanga also suggested that maternal outreach services should serve as an intervention for healthcare professionals to screen and refer to appropriate healthcare professionals as that will help to reduce the number of consultations and hospital admissions [42]. This study’s findings are comparable to those conducted in Australia, emphasizing that outreach programs lessen self-referrals. Therefore, outreach programs should continue in local communities [43]. As suggested in other countries, maternal outreach programs would benefit primary and secondary healthcare facilities, especially in conducting health talks and tracing high-risk pregnant women.

Priority equipment provision was also suggested as a strategy that can assist in providing quality MHC and reduce the MMR, where it was suggested that the department and hospital management should ensure that all the resources, such as birthing beds, are available for safer care. Similarly, a study conducted in Germany has suggested that, in order to improve the quality of maternal and child healthcare as a crucial step toward achieving the SDG, there should be a priority place to ensure the availability of equipment in public healthcare facilities [44]. This finding concurs with recommendations suggested by a study conducted in Tanzania that suggested that with high maternal and child deaths developing globally, the government and other stakeholders should ensure the provision of the necessary equipment to public hospitals and the functionality of that equipment [45]. This study strongly suggests that equipment should be made available in public hospitals all the time because the lack of equipment affects maternal health negatively.

The study participants also suggested that the Department of Health should employ more healthcare professionals, which would serve as a strategy to improve the quality of maternal health services in hospitals. They also highlighted that those retiring, passing away and resigning should be replaced to avoid an increased workload. The findings are compatible with a study conducted in a public hospital in Tshwane, South Africa, that emphasized that more midwives need to be employed because the shortage of midwives was reported to be directly related to the poor provision of quality care because of an increased workload that leads to stress and burnout [46]. Similarly, a study conducted in the Rundu Intermediate hospital and Nyangana district hospital, Kavango East region in Namibia, attested that several studies showed that there is a global shortage of midwives causing significant problems in hospitals and clinics; therefore, it was suggested that more midwives need to be hired to address the shortage of staff in maternal health [47]. This study implies that the government should invest in hiring more midwives in public hospitals for proper support and provision of quality maternal health.

### 4.1. Recommendations

There were various issues related to high maternal deaths, which this study narrowly addressed. Therefore, the following are recommendations for future researchers: research should include primary healthcare facilities and explore a larger scale so that the results can be generalized. Additionally, pregnant inpatients can also be considered to form part of this study. Thus far, there are no studies conducted in SA that aim to explore ENAP’s effectiveness. Additionally, based on the findings of this study, there is a need to re-introduce ENAP so that its effectiveness can be monitored based on healthcare professionals’ views and understanding.

### 4.2. Limitations of This Study

- Some participants (pregnant outpatients) were reluctant to open up, fearing that whatever they said may be used against them.
- The findings of a study from medical doctors working under maternal health services cannot be generalized to a broader population, because a smaller number of doctors were interviewed.
- The duration of the interviews was limited as participants needed to agree to set a proper appointment. They preferred to be interviewed the day the researcher visited the hospital, so it limited some to elaborating their responses as they were on duty.
- Female midwives were more willing to participate than males. Therefore, only female midwives were interviewed and the researcher did not hear the males’ views.

## 5. Conclusions

Even with all strategies launched throughout the districts in SA by the National Department of Health, the standard of maternal health service still needs to improve in the Vhembe district, Limpopo Province. Although healthcare professionals find some strategies effective and helpful, periodic monitoring needs to be performed to determine loopholes and manage them on time. The lack of material resources, shortage of staff, incompetent staff and poor infection control contribute to maternal deaths and poor maternal healthcare services. In acknowledgement of those factors, required resources should be made available all the time; providing continuing in-service training to midwives, launching maternal health outreach services and employing more skilled healthcare professionals will serve as interventions to assist in reducing the MMR and improve the provision of quality healthcare services in the Vhembe district, Limpopo Province as well as assisting in reaching the SDG by 2030. This study recommends the department hire more midwives as those passing on, resigning and retiring are not being replaced, and guarantee that hospitals always have all necessary resources, such as water and birthing beds. Furthermore, the department should ensure that midwives receive emotional and physical support as they experience excessive stress when dealing with childbirth complications with little staff support. Moreover, this study recommends frequent monitoring of the maternal healthcare system to ensure adequate functionality in healthcare facilities. It is imperative that the Department of Health in South Africa takes immediate and substantial measures to oversee and motivate midwives in implementing the skills and protocols they have acquired for managing obstetric emergencies alongside the introduction of ESMOE.

## Figures and Tables

**Table 1:** Biographical information of midwives.

Participants (Codes)	Gender	Age	Hospital	No. of Years Working in a Hospital
P1	Female	52	Hospital E	Two years
P2	Female	29	Hospital E	Eight years
P3	Female	35	Hospital E	Two years
P4	Female	29	Hospital A	Two years
P5	Female	31	Hospital A	14 years
P6	Female	42	Hospital A	Four years
P7	Female	40	Hospital F	Four years
P8	Female	56	Hospital F	16 years
P9	Female	30	Hospital F	Three years
P10	Female	31	Hospital C	Two years
P11	Female	25	Hospital C	Six years
P12	Female	27	Hospital C	11 years

**Table 2:** Biographical information of doctors working in maternal health services.

Participants (Codes)	Gender	Age	Hospital	No. of Years Working in a Hospital
P13	Male	34	Hospital E	Three years
P14	Male	38	Hospital A	Three years
P15	Male	40	Hospital F	Five years
P16	Female	31	Hospital C	Two years

**Table 3:** Biographical information of pregnant outpatients.

Participants (Codes)	Age	Hospital
P17	31	Hospital E
P18	33	Hospital E
P19	24	Hospital E
P20	30	Hospital A
P21	32	Hospital A
P22	23	Hospital A
P23	20	Hospital F
P24	38	Hospital F
P25	26	Hospital F
P26	29	Hospital C
P27	40	Hospital C
P28	31	Hospital C

**Table 4:** Summary of results from data analysis.

Negative Interaction with Midwives	Factors Affecting Maternal Health Services	Views on Existing Strategies/Approaches	Recommended Strategies to Improve Maternal Healthcare and Reduce MMR
Negative attitude Non-empathetic language Frustrations Ill-treatment Lack of information	Lack of material resources Shortage of staff Poor infection control	ESMOE formation CARMMA implementation BANC establishment ENAP approach	Continuing in-service training Launching maternal health outreach services Priority equipment provision Employ more healthcare professionals

## Data Availability

Data are available upon request at tshisikhawe01@gmail.com.

## Public Involvement Statement

No public involvement in any aspect of this research.

## Guidelines and Standards Statement

This manuscript was drafted against the integrated guideline for reporting qualitative studies (COREQ) [10].
